# Enhancing Active Engagement for Dementia Caregivers: A Synthesis of Interventions

**DOI:** 10.1093/geroni/igab046.1075

**Published:** 2021-12-17

**Authors:** Jacqueline Eaton

**Affiliations:** University of Utah, University of Utah, Utah, United States

## Abstract

In a recent meta-analysis of interventions for dementia caregivers, psychoeducational interventions were found to be effective only if they required caregivers to apply knowledge and skills through active engagement. This emphasizes the importance of understanding which intervention components enhance application in order to improve caregiving interventions and the mechanisms by which they work. The purpose of this presentation is to identify and assess elements of active engagement within dementia caregiving interventions. Articles included in this review were published between 2009 and 2018 and identified as psychoeducational dementia caregiving interventions. Each intervention was assessed to describe: 1) how active engagement was defined, 2) the logistics for implementing the active engagement techniques, 3) and the process for evaluating active engagement components. Of 36 articles meeting inclusion criteria, 25 mentioned active engagement components of the intervention. Active components included discussion, problem-solving, practice, role-play, action plans, and homework. Only five articles provided partial descriptions of the active components, five mentioned assessing active engagement, and only one study examined the efficacy of an engagement technique. This demonstrates a significant gap in our understanding of interventions for dementia caregivers. Active engagement enhances outcomes, yet to our knowledge, the specific steps taken to engage caregivers actively and the mechanisms by which these work are unclear. This is a barrier to optimizing active engagement within intervention delivery. Clarifying processes and methods for testing mechanisms of action can further enhance caregiver engagement with interventions.

